# Annonaceous acetogenins mediated up-regulation of Notch2 exerts growth inhibition in human gastric cancer cells *in vitro*

**DOI:** 10.18632/oncotarget.15502

**Published:** 2017-02-18

**Authors:** Yan Li, Jianbin Ye, Zhongbiao Chen, Junjie Wen, Fei Li, Pengpeng Su, Yanqing Lin, Bingxin Hu, Danlin Wu, Lijun Ning, Qi Xue, Hongxiang Gu, Yunshan Ning

**Affiliations:** ^1^ School of Laboratory Medicine and Biotechnology, Southern Medical University, Guangzhou, 510515, PR. China; ^2^ Nanfang Hospital, Southern Medical University, Guangzhou, 510515, PR. China

**Keywords:** annonaceous acetogenins, gastric cancer cell, Notch2, proliferation, apoptosis

## Abstract

**Background:**

Gastric cancer (GC) is a global health problem because of limited treatments and poor prognosis. Annonaceous acetogenins (ACGs) has been reported to exert anti-tumorigenic effects in cancer, yet the mechanism underlying its effects on GC remains largely unknown. Notch signaling plays a critical role in cell proliferation, differentiation and apoptosis. Therefore, it may contribute to the development of GC. This study aims to explore the role of Notch2 in ACGs’ activities in GC cells.

**Results:**

ACGs inhibited GC cells’ viability in a dose dependent manner and led to cell apoptosis and cell cycle arrest in G0/G1 phase with an increased Notch2 expression. Additionally, Notch2 siRNA reduced ACGs-induced cell growth inhibition while Notch2 cDNA transfection did the opposite.

**Materials and Methods:**

ACGs were administrated in GC cells and cell proliferation was assayed by MTS, cell apoptosis and cell cycle were detected by flow cytometry. Additionally, the expression of Notch2 and the downstream target Hes1 were identified by Western blot. Furthermore, Notch2-siRNA transfection and Notch2-cDNA were performed to investigate the role of Notch2 in the antitumor effect of ACGs. Conclusions: Up-regulation of Notch2 by ACGs is a potential therapeutic strategy for GC.

## INTRODUCTION

Gastric cancer (GC) is one of the most common malignancies in global scale. It also acts as one of major causes of cancer-related death. Many current available treatments for gastric cancer, such as surgery and chemotherapy, are less optimal, and the prognosis of GC is rather poor. This is because of lacking complete understanding in the mechanisms for GC. Therefore, it is critical to explore alternative treatments and develop more effective therapies.

One promising candidate for chemopreventive and chemotherapeutic drugs is traditional Chinese medicine. A variety of novel natural compounds, such as podophyllotoxin, paclitaxel, camptothecin, and vinblastine, have been isolated and applied as agents for cancer therapies. Annonaceae, belonged to soursop family or custard apple family, with approximately 120 genera and 2,000∼2,200 species, is the largest family in Magnoliales. The whole plants, seeds or fruits of Annonaceous plants have been widely explored as popular traditional medicines for the treatment of pain, diarrhea, fever and hypotension. Annonaceous acetogenins (ACGs) is a group of fatty acid derivatives which are isolated from Annonaceae plants [1]. In 1982, when the first ACGs compound, uvaricin, was isolated, its excellent in-vivo anti-leukemia activity aroused widespread interest amongst medicinal chemicals and natural products to isolate and identify this class of compounds [2]. The potentially useful applications of ACGs were exhibited, such as cytotoxicity, anti-microbial, anti-malarial, anti-feedant, anti-viral, anti-tumoral, anti-helminthic, pesticidal, and immunosuppressive activities [2, 3]. The powerful cytotoxicity against tumor made ACGs as another “tomorrow anticancer star” after paclitaxel. However, the antitumorigenic mechanism of ACGs is still unclear.

Notch signaling, a highly conservative signaling pathway, is involved in a variety of cellular processes including cell proliferation, differentiation, apoptosis, fate and survival rate. Additionally, the abnormity of Notch signaling is associated with oncogenesis [4–6], which usually manifests as abnormality of Notch signal components like ligands, receptors, and downstream proteins. The Notch signaling in mammals consists of four receptors (Notch 1-4) and five ligands Delta-like ligand 1/3/4 (DLL1/3/4) and Jagged (1/2) [7]. After the interaction of the receptors to their ligands, the γ-secretase cleaves the transmembrane domain of Notch receptor to release the intracellular domain of Notch receptor (NICD), which was translocated into the nucleus as a transcriptional coactivator, regulating the expression of target genes, such as the hairy enhancer of split (Hes) and Hes-related (Hey) family [7]. Clearly, activated Notch receptor can play a tumor suppressive or an oncogenic role depending on the tumor type and cellular context [8, 9]. For example, Notch acts as a tumor suppressor in squamous cell carcinoma of skin [10], cervical uterus [11], hepatocellular carcinoma and neuroendocrine tumors of lung and gastrointestinal tract [12]. On the contrary, Notch may have oncogenic impact on breast cancer [13], colorectal cancer [14], neuroblastoma [15], and lung cancer [16]. At present, a number of researches have been conducted to explore the association between Notch signaling pathway and GC in human. However, the function of components of Notch pathway in GC is still controversial because of its indicated different even opposite effects [17]. On one hand, Notch1 has been detected to be expressed in normal gastric mucosa as well as most GC cell lines [18], on the other hand, some data illustrated that there was no expression in normal gastric mucosa [17]. The meta-analysis by Du X et al. revealed that Notch1 was expressed in both GC tissues and normal mucosa, while significantly higher expression was found in cancer tissues than in normal tissues, suggesting the activation of Notch1 in GC [19]. This is consistent with the oncogenic role of Notch1 in many solid malignancies. As for Notch2, Sun et al. proved that Notch2 was overexpressed in GC. Additionally, co-expression and nuclear co-translocation of Notch2 and its downstream target protein Hes1 were seen to be more frequent than Notch1 both *in vitro* and *in vivo* in GC [17], suggesting that Notch2 signal pathway would be more important in GC carcinogenesis and progression. Tseng et al. showed that the activated Notch2 would promote both cell proliferation and xenografted tumor growth of GC cells through cyclooxygenase-2 [20]. Conversely, Guo et al. showed that Notch2 as a tumor suppressor gene could inhibit cell invasion of human GC [21]. No doubt that, it is necessary to detect potential roles of Notch signaling and the activation patterns in different tumor types without any initial impression.

To date, the role of Notch2 signal pathway in the antitumor activity of ACGs has not been investigated. In this study, ACGs was administered in GC cells to detect the cellular process affected by this compound and whether it played a tumor suppressor role through the regulation of Notch2.

## RESULTS

### The expression of Notch2 was increased or decreased in GC cell lines

**Figure 1:**
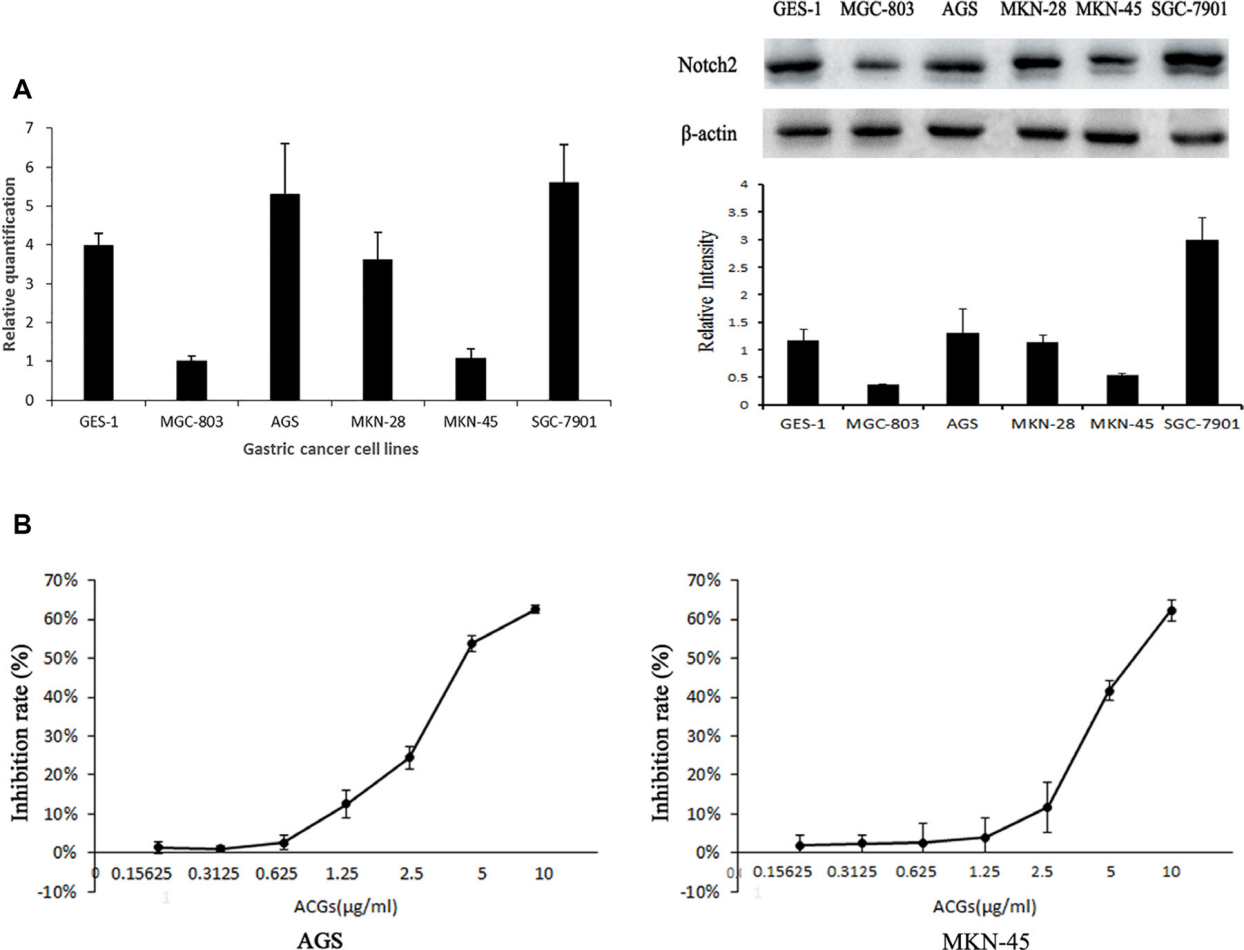
(**A**) Comparison of Notch2 expression level at mRNA and protein level among GC cell lines. Left: Expression of Notch2 gene was detected by real-time fluorescence quantitative-PCR (RFQ-PCR), *n* = 3. Right: Expression of Notch2 protein was detected by western blot, *n* = 3. (**B**) The inhibition rate was calculated as the following equation: inhibition rate (%)=(1-OD of ACGs treatment group/ OD of control group) ×100%. The half maximal inhibitory concentration (IC 50) is a measure. The solvent control was 0.1% DMSO. The results are expressed as the means ± SEM, *n* = 6.

In order to evaluate the possible role of Notch2 in gastric carcinogenesis, we screened a panel of 5 GC cell lines for the relative expression of Notch2 at mRNA level by quantitative real-time PCR and at protein level by western blot. Compared with normal gastric mucosa cell line GES-1, Notch2 expression varied quantitatively with GC cell lines. Notch2 expression was higher in AGS and SGC-7901 and lower in MGC-803, MKN-28 and MKN-45 (Figure 1A), which was consistent with the published results. IC50 of ACGs to cells for 24 h was assayed by MTS. The IC50 of AGS and MNK45 was approximately close with 5.02 ug/mL and 6.25 ug/mL respectively (Figure 1B). Then AGS (high Notch2 expression) and MKN-45(low Notch2 expression) were selected to perform in the following experiments.

### Cell growth inhibition by ACGs in a dose-dependent manner

**Figure 2:**
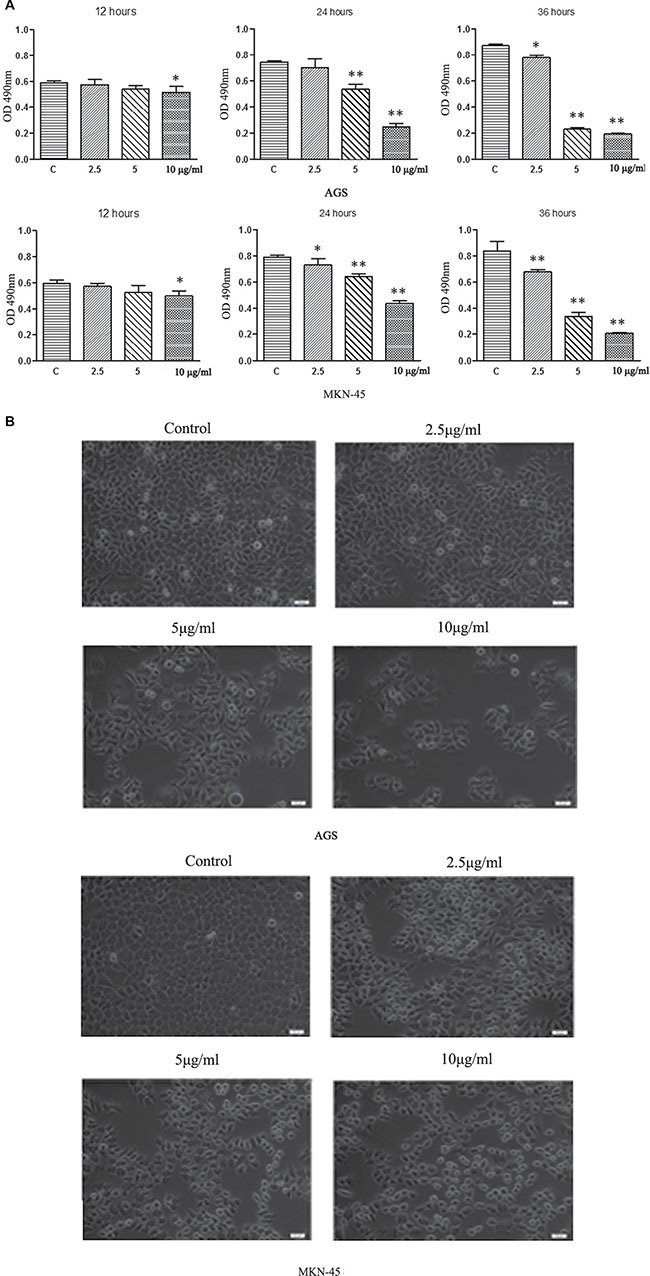
(**A**) ACGs inhibited AGS and MKN-45 cells growth in a dose and time-dependent manner. AGS and MKN-45 cells were treated with 2.5 μg/ml,5 μg/ml, and 10 μg/ml ACGs for 12 h, 24 h, and 36 h respectively. Cell proliferation was tested by MTS assay. Data represented mean± SEM, *n* = 6. The statistical significant was confirmed compared with control group. *P < 0.05, **P < 0.01. (**B**) Effects of ACGs administration on GC cell morphology. Cells were treated with ACGs at the concentrations 2.5, 5 and 10 μg/ml for 36 respectively. Cell morphology was observed under an inverted phase contrast microscope and images were obtained. Significant cell shrinkage and a decreased cellular attachment rate were observed in the ACGs-treated group.

To investigate whether ACGs affects the viability of GC cells, cells were treated by ACGs for 12, 24, 36 h with 2.5 μg/mL, 5 μg/mL, and 10 μg/mL respectively, and then the growth of cells was measured by MTS. The inhibition of cell growth by ACGs showed an increasing trend in a dose-dependent manner in 24 h group and 36 h group in both GC cell lines (Figure 2A). In addition, microscopy images showed that ACGs treatment increased significant cell shrinkage and decreased the cellular attachment in comparison with the control group (Figure 2B).

### Cell apoptosis induced by ACGs

**Figure 3:**
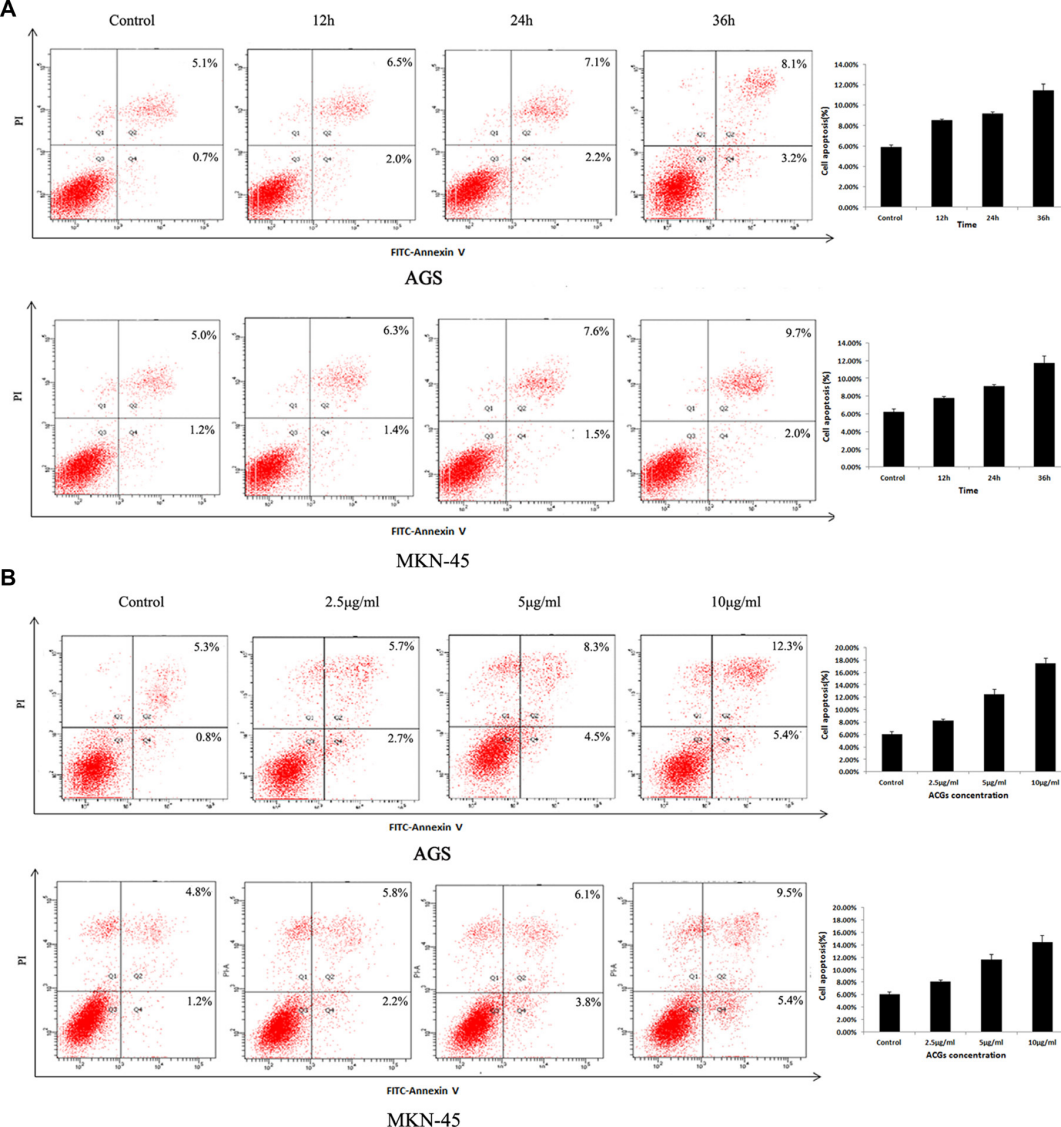
Effects of ACGs administration on the apoptosis in AGS and MKN-45 cells (**A**) GC cells were treated with 5 μg/mL ACGs for 12 h, 24 h, 36 h respectively, stained with Annexin V/PI and analyzed by flow cytometry. (**B**) GC cells were treated with 2.5, 5 and 10 μg/ml ACGs for 36 h respectively, stained with Annexin V/PI and analyzed by flow cytometry. Representative flow cytometric analyses of apoptosis are shown. Four subpopulations and their fractions are indicated: normal cells (lower left), dead cells (upper left), early apoptotic cells (lower right), and late apoptotic cells (upper right). The apoptotic indices are expressed as the number of apoptotic cells/the total number of counted cells ×100%.

In order to explore whether the cell growth inhibition by ACGs was accompanied by the induction of apoptosis, the effect of ACGs on GC cell death was examined. After administration with 5 μg/mL ACGs for 12 h, 24 h, 36 h respectively, cells were stained with Annexin V/PI and analyzed by flow cytometry. The effect of induction of ACGs was detectable in all three time points compared with the control group (Figure 3A). Furthermore, GC cells were induced apoptosis by ACGs in a dose-dependent manner after cells were treated with different concentrations of 2.5, 5 and 10 μg/ml for 36 h (Figure 3B).

### Cell cycle was arrested in G0/G1 phase by ACGs

**Figure 4:**
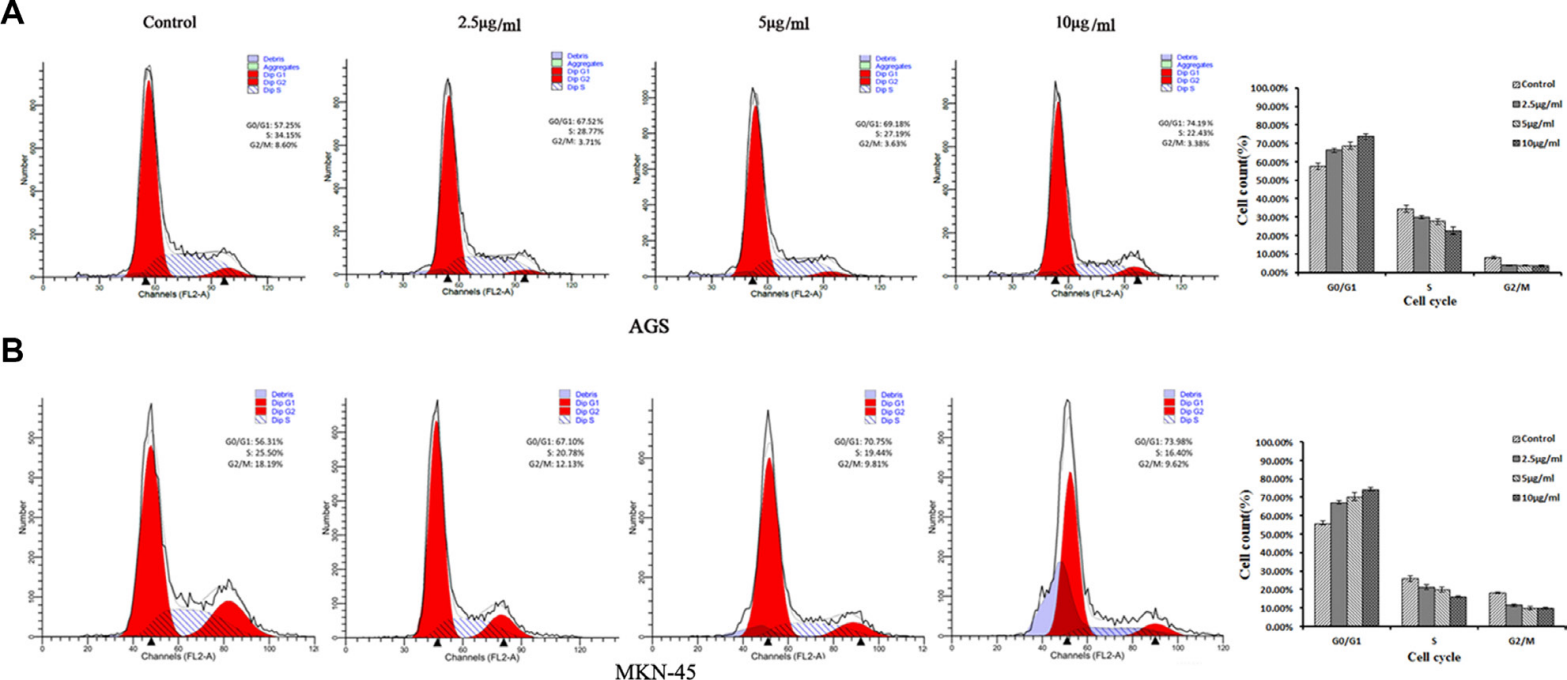
Cell cycle analyses of ACGs-treated AGS and MKN-45 cells by flow cytometric GC cells were treated with the concentration of 2.5, 5 and 10 μg/ml ACGs for 36 h, respectively. Then cells were harvested, stained with PI and subjected to flow cytometry for analyzing cell distribution at each phase of the cell cycle.

In order to explore whether the cell growth inhibition by ACGs was accompanied by cell cycle arrest, the effect of ACGs on GC cell cycle was examined. After the treatment of ACGs with 2.5, 5 and 10 μg/ml for 36 h respectively, the course of cell cycle was arrested in G0/G1 phase compared with the control group. The ratio of G0/G1 phase of AGS cell was increased to 67.52%, 69.18% and 74.19%, respectively. The ratio of G0/G1 phase of MKN-45 cell was increased to 67.10% 、 70.75% and 73.98%, respectively (Figure 4). These results suggested that ACGs arrested the cell cycle of G0/G1 phase in GC cells.

### ACGs increased Notch2 expression and decreased Hes-1 expression

**Figure 5:**
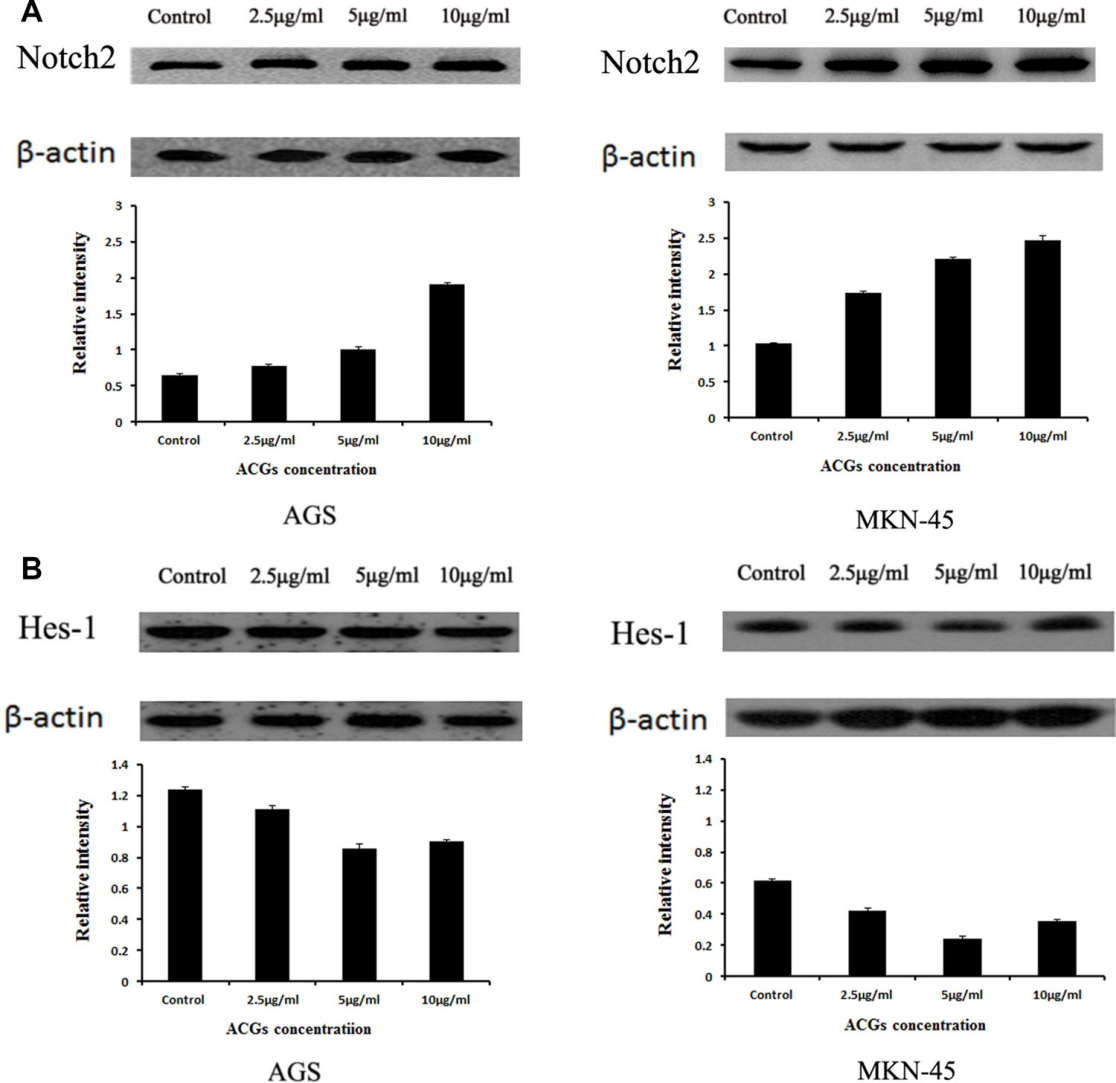
The expression of Notch2 and Hes1 in AGS and MKN-45 cells after ACGs treatment GC cells were treated with the concentration of 2.5, 5 and 10 μg/ml ACGs for 36 h, respectively. The expression of Notch2 and Hes-1 protein was quantified by Western blot and compared with internal control β-actin. Control cells were treated with 0.1% DMSO. Each experiment was repeated three times. (**A**) The expression of Notch2. (**B**) The expression of Hes-1.

Based on above results, we wanted to clarify a possibility that the effect of ACGs on Notch2 signaling. Therefore, the expression of Notch2 was examined after GC cells were respectively treated with 2.5, 5 and 10 μg/ml ACGs for 36h. The expression of Notch2 was increased in all ACGs-treated cells compared with control group (Figure 5A). However, ACGs decreased the expression of its down-stream target, Hes-1 (Figure 5B). The levels of Hes-1 did not correlate with the level change of the Notch2 receptor. This discrepancy may be attributed to the complicated cellular regulation of Notch gene expression.

### siRNA mediated-down-regulation of Notch2 expression reduced ACGs-induced cell growth inhibition in GC cells

**Figure 6:**
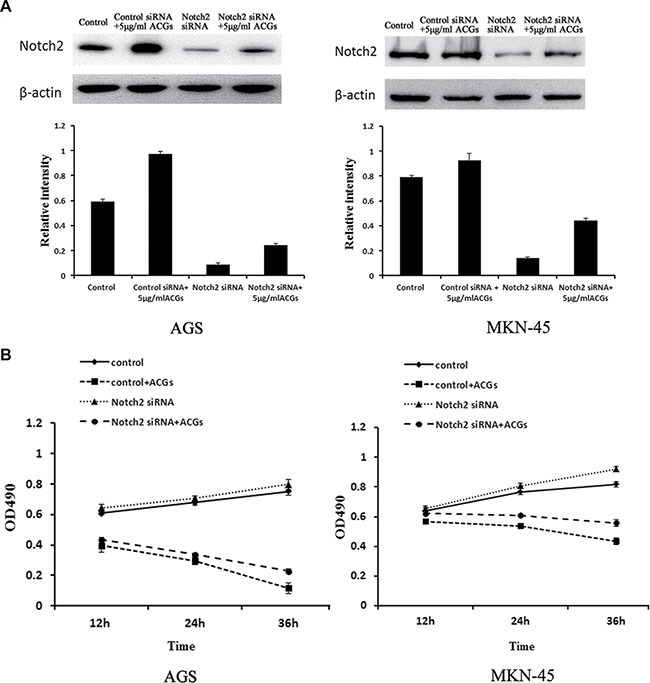
GC cells were transfected by Notch2-siRNA for 24 h and then treated with 5 μg/ml ACGs for 24 h The control cells were transfected by control Notch2-siRNA. Each experiment was repeated three times. (**A**) Top: The expression of Notch2 was detected by Western blot compared with internal control β-actin; Each histogram indicates the relative band intensity (**B**) The cell growth was detected at 12, 24 and 36 h by MTS after ACGs treatment.

To further study the tumor suppressive role of notch2, Notch2-siRNA was conducted to evaluate the effect of down-regulation of Notch2 signal pathway on the antitumor activity of ACGs *in vitro*. As shown in Figure 6, the expression of Notch2 was dramatically decreased by Notch2-siRNA transfection and the growth of cells transfected by Notch2-siRNA was considerably faster than that of non-transfected cells. In addition, the combination of Notch2-siRNA transfection and ACGs treatment slightly inhibited cell growth compared with ACGs-treated alone, suggesting Notch2 siRNA reduced ACGs-induced cell growth inhibition to a certain degree.

### Over-expression of Notch2 by cDNA transfection promoted ACGs-induced cell growth inhibition in GC cells

**Figure 7:**
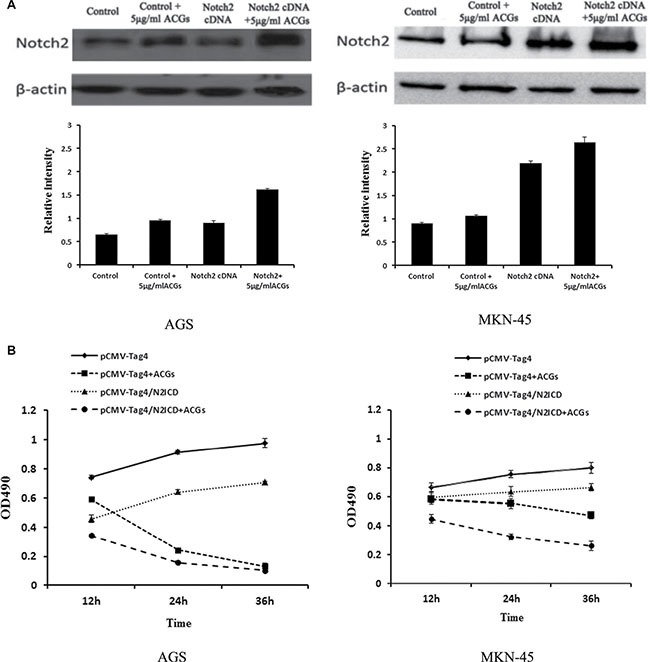
GC cells were transfected by Notch2-cDNA for 24 h and then treated with 5 μg/ml ACGs for 36 h The cells were transfected by pCMV-Tag4 vector as control. Each experiment was repeated three times. (A) Top: The expression of Notch2 was measured by western blot compared with internal control β-actin; each histogram indicates the relative band intensity. (B) The cell growth was detected at 12, 24 and 36 h by MTS after ACGs treatment.

In order to further determine the tumor suppressive effect of Notch2, GC cells were transiently transfected with Notch2 coding sequence prior to ACGs treatment. As shown in Figure 7, Notch2 cDNA transfection induced up-regulation of Notch2 protein confirmed by western blot, and the growth of GC cells transfected with Notch2 cDNA was considerably slower than that of non-transfected cells. Moreover, this combination of over-expression of Notch2 and ACGs treatment strongly inhibited cell growth compared with ACGs-treated alone, suggesting the combination promoted ACGs-induced cell growth inhibition to a certain degree.

## DISCUSSION

Annonaceous acetogenins (ACGs), which consists of a series of natural products isolated exclusively from Annonaceae species, exhibits significant cytotoxicity and potent anticancer effect since 1900s [22]. There are more evidences illustrating the anti-tumor effect of ACGs on different cancers in the last 20 years [23]. AA005, which mimics ACGs, induce colorectal cancer cell death [24] and leads to the growth inhibition and autophagy in colon cancer cells [21]. Similarly, Desacetyluvaricin, one of ACGs components, inhibits the growth of colorectal cancer cell line SW480 [25]. Thiophene-3-carboxamide analogue of ACGs strongly inhibits the growth of human lung cancer cell line NCI-H23 in the xenograft mouse without critical toxicity [26]. The interactions among acetogenins in Graviola (Annona muricata) leaves and flavonoids synergistically confer protection against prostate cancer [27]. Three new ACGs with mono-tetrahydrofuran rings from graviola fruit (Annona muricata) demonstrate cell growth inhibition in human prostate cancer PC-3 cells [28] in a similar manner. In the current study, MTS assay and flowcytometry analysis show that ACGs inhibited cell growth and induce apoptosis in time and dose –dependent manner. Additionally, cell cycle analysis indicated that ACGs induced GC cells accumulation in G0/G1 phase in a dose-dependent manner. Nevertheless, the exact mechanisms of ACGs-induced these effects remain obscure.

It is well known that Notch signal pathway is involved in cell proliferation, differentiation, apoptosis, cell fate and maintenance of stem cells. Furthermore, Notch signal pathway can exhibit tumor-suppressive or oncogenic functions and the regulatory mechanisms of Notch signaling are complicated and the biologic effects can exert opposite functions in time and context-dependent manners [29, 30] even in the same tumor type [7]. For instance, a tumor-suppressive function has been found in skin and liver cancers, an oncogenic role in NSCLC [7, 31] and a dual role in the same tumor type of colorectal and pancreatic cancers [32, 33]. In addition, phenotypes exhibited by the combined activation of multiple Notch signaling may be different from either pathway alone [34]. For example, Notch1 and Notch2 receptors work oppositely on the survival of malignant mesothelioma cells [35]. Notch2 receptor also shows the contrary functions of endothelial cells growth compared with Notch1 and Notch4 [36].

Mounting evidences suggested that Notch signaling may participate in the regulation of GC progression [17, 20, 37–39]. The intracellular domains of Notch1 and Notch2 receptors (N1ICD and N2ICD, respectively), promoted cell proliferation and xenografted tumor growth of human stomach adenocarcinoma SC-M1 cells [20, 37]. The colony formation, invasion, migration, and wound-healing abilities of SC-M1 cells were increased by N2ICD expression, whereas these abilities were decreased by Notch2 knockdown. Similarly, Notch2 knockdown suppressed cancer progressions of AZ521 and AGS GC cells [20]. In contrast, the findings of Bauer et al’s study ascribed a tumor-suppressive role to Notch signal pathway in GC, in which the close relationship of high Notch1 and Notch2 expression was associated with early tumor stages [40]. This result was essentially consistent with a functional analysis of Notch2 in GC cell line MKN-45, which indicated that down-regulation of Notch2 by siRNA increased tumor cell invasion [21]. Additionally, co-expression and nuclear co-translocation of Notch2 as well as the downstream target protein Hes-1 were detected to be more frequent than Notch1 both *in vitro* and *in vivo* in GC [17], suggesting that Notch2 mediated signal pathway would be more vital in the tumorigenesis and progression of GC. Consequently, Notch2 signal pathway is clearly involved in the pathogenesis of GC, which is intended to address whether ACGs may exert its antitumor effect through Notch2 pathway.

In the current study, compared with normal gastric mucosa cell, Notch2 expression was higher in AGS and SGC-7901 and lower in MGC-803, MKN-28 and MKN-45 confirmed by Q-PCR. AGS and MKN-45 were selected to perform the subsequent experiments. We demonstrated for the first time that ACGs treatment increased Notch2 expression in a dose-dependent manner in GC cells and induced apoptosis and arrested cell cycle in G0/G1, suggesting regulation of Notch2 signaling may be the antitumorigenic mechanism of ACGs. Furthermore, loss-function by Notch2-siRNA and gain-function by over-expression of Notch2-cDNA transfection in GC cells were carried out prior to ACGs treatment. Notch2-siRNA together with ACGs treatment reduced cell growth inhibition to a greater degree compared with ACGs alone. Conversely, the over-expression of Notch2 by cDNA transfection promoted ACGs-induced cytotoxity to a certain degree compared with ACGs alone. Taken together, we strongly believe that up-regulation of Notch2 by ACGs is associated with cell growth inhibition and linked mechanistically to apoptotic processes as well as cell cycle G0/G1 arrest.

However, the levels of Hes-1 in GC cells did not correlate with the level change of Notch2 receptor. This discrepancy may be attributed to the complicated cellular regulation of Notch gene expressions. The complicated inter-relationship among the 4 Notch receptors in the regulation of gene expression and cell function remain elusive. Previous studies have demonstrated that the activation of one Notch receptor can repress or induce the other Notch signaling [41, 42]. Moreover, each of 4 Notch receptors may have distinctly selective target sequence [43, 44] and play a differential biological function. Transcription factor Hes-1, a key repressor of its own promoter by a negative feedback loop, has a very short half-life [45]. Consequently, the levels of Hes-1 might not directly reflect the activation of Notch2 receptor.

From what has been discussed above, ACGs treatment in GC cells *in vitro* resulted in enhanced cell proliferation inhibition, apoptosis, G0/G1 arrest and Notch2 expression. In fact, each of these properties positively correlated with the dose of ACGs, with more significant effects at higher doses. Thus, ACGs stand a position that might inhibit proliferation and induce apoptosis and G0/G1 arrest through directly or indirectly activating Notch2 expression. The combined treatment of GC with ACGs and Notch2 signaling might exhibit synergistically therapeutic potential than with higher doses of drug alone. It is also possible not only to reduce drug adverse-effects, but also to offer a novel and efficient therapeutic strategy for the treatment of GC in the future. Further investigation is needed to explore the mechanisms of which how ACGs activates Notch2 signaling and how these changes lead to increase cell proliferation inhibition and apoptosis.

To our knowledge, this is the first investigation regarding the effect of ACGs on GC cells through Notch2 signaling pathway. As demonstrated in the present study, the molecular mechanism by which ACGs exerts its inhibitory effects on GC cells through Notch2 will open up a new era for developing novel therapeutic strategies. More importantly, the up-regulation of Notch2 by ACGs may be a potential strategy for chemosensitization of metastatic GC cells to standard therapeutics. Nevertheless, further in-depth studies together with preclinical animal studies are needed to evaluate the cause-and-effect relation of Notch2 regulation as well as ACGs-induced the inhibition of cell growth and apoptosis in GC to determine this novel hypothesis in future clinical trial.

## MATERIALS AND METHODS

### Annonaceous acetogenins

**Figure 8:**
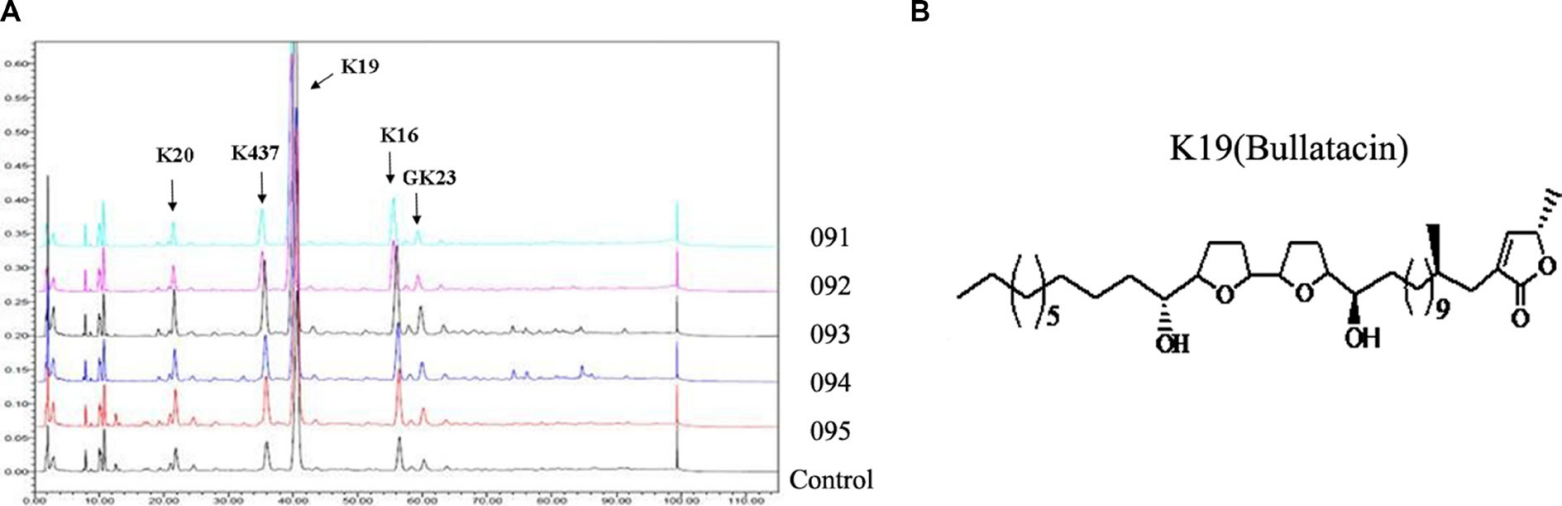
(**A**) HPLC fingerprint chromatograms determined at 210 nm for five batches of total annonaceous acetogenins. (**B**) The structure of Bullatacin.

Annonaceous acetogenins (ACGs) were kindly provided by Dr. Xiangtao Wang (Institute of Medicinal Plant Development, Chinese Academy of Medical Science & Peking Union Medical College). The major components of ACGs in this batch (No. 091) were list in Supplementary Table 1 and K19 (43.124%) was the main component named Bullatacin. HPLC finger print chromatogram was determined at 210 nm for five batches (No.091, 092, 093, 094, 095) of total ACGs (Figure 8A) and K19 (Bullatacin) was still the main component of all batches. The structure of Bullatacin was shown in Figure 8B.

### Cell lines and culture

GC cell lines including AGS, MKN-28, MKN-45, SGC-7901, MGC-803and normal gastric mucosa cell GES-1 were cultured in high-glucose DMEM medium (Hyclone, USA) containing 10% fetal bovine serum (Zhejiang Tianhang Biological Technology Co., Ltd, China), 100 U/mL penicillin and 100 U/mL streptomycin (in a humidified atmosphere of 5% CO2 air at 37°C and medium changed 24 h). Cells were passaged when the confluence was about 90%. Cells were washed with PBS (pH 7.2-7.4) and detached with 0.25% trypsin (Hyclone, USA) for 2-3 min. Then the complete medium was used to wash cells. Cells were used for experiments at logarithmic growth phase.

### RNA extraction and real-time PCR

Total RNA from cultured cells was extracted using the TRIzol method (Invitrogen) and was reverse-transcribed by PrimeScript RT reagent Kit With gDNA Eraser (TaKaRa, Tokyo, Japan) according to manufacturer’s instruction. Briefly, real-time PCR was performed for forty cycles to amplify N2ICD/Notch-related genes (denaturation at 95°C for 5 s, annealing and extension at 60°C for 34 s) in triplicate using PrimeScript II Reverse Transcriptase (TaKaRa, Tokyo, Japan) detected y an ABI PRISM Stepone Plus Sequence Detection System (Applied Biosystems 7500, Foster City, CA, United States) following the manufacturer’s instructions. To test gene expression, relative quantitation of target gene expression was determined by ΔΔCt (threshold cycle) method. ΔCt is the difference between Ct of target mRNA and Ct of internal control for each group. The primers for real-time PCR were listed in Supplementary Table 2.

### MTS colorimetry

Cells (4000 cells/mL) were plated into a 96-well plate with 100 μL suspension per well and cultured for 24 h at 37°C and 5% CO2. ACGs powder was dissolved in dimethyl sulfoxide (DMSO) as 10 mM stock solution. ACGs solution was sterilized with 0.22 μM membrane filtration, aliquoted and then stored at -20°C. For use, stock solution was thawed and diluted with high-glucose DMEM in a series of final concentrations and the final concentration of DMSO in the ACGs solution was < 1%. Solution with ACGs (0.15625 ug/ml∼10 ug/ml) was administered 100 μL per well to different wells with 0.1% DMSO as negative control group. Each of groups comprised 3 sample wells. After ACGs was administered in GC cells, cell viability was assessed with Cell Titer 96^®^ Aqueous One Solution Cell Proliferation Assay (MTS, Promega, USA). Briefly, 20 μL of Cell Titer 96^®^ Aqueous One Solution was added to each well. Following 1h in culture, optical absorbance (A) at 490 nm was detected by a microplate reader (Model 680, BIO-RAD, USA). The average of the absorbance values of all wells was used to calculate the inhibition rate of proliferation: inhibition rate = (1-absorbance of experimental group/ absorbance of control group) × 100%. Then the half maximal inhibitor concentration (IC50) value was deduced.

### Flow cytometry for cell apoptosis

To further explore the molecular mechanism involved in ACGs inhibition of GC cells proliferation, cell apoptosis was evaluated using FITC-Annexin V/PI staining kit. After GC cells were treated 2.5 μg/ml, 5 μg/ml, 10 μg/ml ACGs respectively for 36h with 0.1% DMSO as control, the cells were harvested and washed with PBS, incubated with fluorescein-conjugated Annexin V and PI for 15min and then analyzed by FACScan flow cytometer equipped with the FACStation data management system and cell Quest software (Becton Dickinson, San Jose, CA, USA).

### Flow cytometry for cell cycle

The flow cytometry was applied to investigate the cell cycle perturbation of cells after ACGs treatment. In brief, 1 × 105 cells were incubated with DMEM medium without FBS for 24 h and treated with indicated doses (2.5 μg/mL, 5 μg/mL, 10 μg/mL) of ACGs, respectively. Cells were then harvested, PBS washed amd fixed in ice-cold 70% ethanol, stored at 4°C overnight and spinned down at 2000rpm for 5min next day. After being washed once with PBS, cell pellets were collected and suspended in 50 ug/ml PI reagent (5 ug/ml RNase, 50 ug/ml propidium iodide, 0.1 mM EDTA, 0.1% Triton-X 100, pH = 7.4) in dark at 4°C for 30min. The determination of DNA content was measured by Beckman Coulter Flow Cytometer (Miami,FL, US). The proportion of cells at each phase in cell cycle (G0/G1, S, G2/M) was scored by MultiCycle software (Phoenix Flow Systems, San Diego, CA, US). Apoptotic peak was also determined from the cell cycle pattern. Each experiment was conducted in three times.

### The construction of recombinant eukaryotic expression vector pCMV-Tag4/N2ICD

The human Notch2-cDNA fragment was amplified by using a forward primer 5′-ATTTGCGGCCGCGCCA

TGCGAAAGCGTAAGC-3′ as well as a reverse primer 5′- CCGCTCGAGCGCATAAACCTGCATGTTGTTGTGT -3′, confirmed by sequencing, and then digested by Not I and Xho I-. The 2.3-kb fragment was purified using a PrimeScriptTM One Step RT-PCR Kit Ver.2.0 (Takara, Japan) and was ligated to a Not I –Xho I-digested pCMV-Tag4 vector DNA (Clontech, Mountain View, CA, United States) to construct Notch2/pCMV-Tag4. Positive clones were further verified by Not I -Xho I digestion, sequencing and PCR.

### Western blotting

Cell samples were homogenized in RIPA lysis buffer (Biosharp, China). Total protein extracts were prepared and concentrations were measured using Bradford protein assay kit. Then protein was separated on 10% polyacrylamide gels and transferred electrophoretically to nitrocellulose membranes. Membranes which were blocked with 5% non-fat dry milk in TBS for 1.5h with the following antibodies: anti-Notch2(1:1000), anti-Hes-1(1:1000), anti-βactin(1:1000). were incubated at 4°C with antibodies to Notch2, Hes-1, β-actin(1:1000, Abcam) and followed by a secondary antibodies (1:500) at room temperature. Washed with TBST three times was required after each step. The bands of proteins were confirmed by luminescent visualization using an ECL western blotting Detection System (Bio-Rad, Hercules, CA, USA) and quantified using the Quantity One software package (West Berkeley, CA, USA).

### The transfection of Notch2-small interfering RNA (siRNA)

Notch2-siRNA (sc-40135, Santa Cruz Biotechnology, Inc) is the target-specific 19-25 nt siRNA designed to knock down human Notch2 gene expression. For siRNA transfection, GC cells were seeded into 6-well plates to be grown to sub-confluency and transiently transfected with negative control siRNA (sc-44236, Santa Cruz Biotechnology, Inc) or Notch2-siRNA at 50 nM for 24 h using Lipofectamine 2000 (Invitrogen, San Diego, CA, USA) according to the manufacturer’s instruction. The next day, the medium was changed to fresh medium with or without ACGs (5 μg/ml), and the cells were continually cultured for 24 h. The proteins were extracted from cells to measure Notch2 expression by western blot and the cell viability was detected by MTS assay.

### Overexpression of Notch2 by cDNA transfection

Transient over-expression of Notch2 was conducted using Lipofectamine 2000 (Invitrogen) following the protocol suggested by the manufacturer. GC cells were transiently transfected with Notch2/pCMV-Tag4 or vector alone (pCMV-Tag4) for 24 h and then were treated with 5 μg/mL ACGs for 24 h. The proteins were extracted and verified by Western blot. Moreover, the cell growth was detected by MTS assay according to the procedures described above.

### Statistical analysis

Data are shown as mean ± standard deviation (x ± s). Statistical analysis were performed on SPSS17.0. Pair-wise comparisons (SNK-q test method), single factor analysis of variance (one-way ANOVA) and inter-group mean values were used on above results. The analyses were performed using two-sided tests. *P* < 0.05 was considered statistically significant.

## SUPPLEMENTARY MATERIALS TABLES


