# Supplementary material for: Annonaceous acetogenins mediated up-regulation of Notch2 exerts growth inhibition in human gastric cancer cells in vitro
Source: Oncotarget. 2017 Feb 18;8(13):21140–52. doi: 10.18632/oncotarget.15502 (PMC5400572; doi:10.18632/oncotarget.15502)
Supplement: Supplementary file 1 [file oncotarget-08-21140-s001.pdf]

Annonaceous acetogenins mediated up-regulation of Notch2 exerts growth inhibition in human gastric cancer cells *in vitro*

Supplementary Materials

Supplementary Table 1: Content percentage of the 5 major components

Major components	Percentage
K20	0.142%
GK23	1.355%
K437	8.929%
K19(Bullatacin)	43.124%
K16	12.145%
Total content	65.695%

Supplementary Table 2: Primers for real-time PCR

Name	Sequence (5′-3′)
N2ICD-F	AAAAATGGGGCCAACCGAGAC
N2ICD-R	TTCATCCAGAAGGCGCACAA
β-actin-F	TGGCACCCAGCACAATGAA
β-actin-R	CTAAGTCATAGTCCGCCTAGAAGCA
